# Association between Plasma Urotensin II and Risk of Hypertension: Findings from a Prospective Study

**DOI:** 10.1155/2020/3284769

**Published:** 2020-01-30

**Authors:** Huijian Xie, Xinya Wang, Yan He, Daoyi Huang, Xinyuan Qu, Yanbo Jiang, Yan An, Shaoyan Zhang, Mingzhi Zhang

**Affiliations:** ^1^Department of Epidemiology, School of Public Health and Jiangsu Key Laboratory of Preventive and Translational Medicine for Geriatric Diseases, Medical College of Soochow University, Suzhou 215123, China; ^2^Center for Disease Prevention and Control of Pudong New District, Shanghai 200120, China; ^3^Macrostat (China) Clinical Research Co., Ltd., Shanghai 200120, China; ^4^Department of Radiology, Shanghai East Hospital, Shanghai 200120, China

## Abstract

Up to date, human urotensin II (UII) is the most potent vasoconstrictor in mammalian animals. To explore whether UII played an important role in the development of hypertension, we conducted a prospective study in Changshu city, China. The baseline investigation was carried out in 2007, and the first follow-up investigation was conducted in 2013. From the participants, we randomly obtained 2000 normotensive subjects aged 40 years and older without any severe disease at baseline and examined plasma UII and endothelin-1 (ET-1) with their blood samples at baseline. Logistic models were used to analyze the association between baseline UII, baseline ET-1, and newly occurring hypertension. In 1,819 subjects with complete data, 723 subjects developed into hypertensive in about five years. After adjusting some potential confounders, the odds ratio (95% confidence interval) for risk of hypertension comparing the highest with the lowest quartile of baseline UII was 0.888 (0.689–1.144). The role of UII in the development of hypertension was not found in the current study; therefore, further research studies should be conducted to explore the relationship between UII and hypertension.

## 1. Introduction

Hypertension, the leading cause of morbidity and mortality of cardiovascular diseases (CVD), has become a global public health challenge [1]. Although a lot of studies have been conducted to explore the risk factors of hypertension, the etiology of hypertension is still obscure.

Urotensin II (UII) is a cyclic peptide initially isolated from the urophysis of the goby fish based on its potent vasoconstrictor effect [2], and then it was cloned from humans. UII is considered as the most potent vasoconstrictor, and its vasoactive activity is even more potent than endothelin-1 (ET-1) [3, 4]. Because of the inconsistent results of the published studies [5–8], the association between UII and hypertension has not been confirmed yet. Furthermore, most of these studies are case-control studies, which are weak to explore the causal relationship between UII and hypertension.

Moreover, some experimental researchers have found that UII, angiotensin II (Ang II), and ET-1 may have a potential interaction in modulating the cardiovascular effects [9–11]. And a case-control study also found that increased plasma levels of UII and ET-1 in patients with coronary heart disease (CHD) and UII and ET-1 were positively correlated [12]. All these studies indicated that UII may play a role in the development of hypertension. So we conducted the prospective study to verify the effect of UII in the development of hypertension.

## 2. Materials and Methods

### 2.1. Study Populations

A large-scale cohort on CVD has been built in rural communities of Changshu city, Jiangsu province, China since 2007. From 2007 to 2008, we conducted a baseline investigation. A total of 20,343 participants aged 18 years and older were enrolled in the study. The first follow-up investigation was held in 2013. For the current study, we randomly selected 2000 normotensive subjects aged 40 years and older according to the baseline characteristics and examined the plasma UII and ET-1 levels with their baseline blood samples. The chosen individuals have also been required no coronary heart disease, stroke, chronic kidney diseases, tumors, chronic obstructive pulmonary diseases, or peripheral artery diseases. Finally, 1819 subjects with complete data were included in the current study. The detailed description of methods for study participant recruitment and baseline data collection has previously been done [13].

This study was approved by the Soochow University Institutional Ethics Review Board and was following the guidelines of the Declaration of Helsinki. All participants provided written informed consent.

### 2.2. Data Collection

The data on demographic characteristics, lifestyle risk factors, family history of CVDs, and medical history were obtained using a standard questionnaire administered by the trained staff. Cigarette smoking was defined as ever having smoked at least 100 cigarettes. The information regarding the amount and type of alcohol consumed during the past years was collected, and alcohol consumption was defined as drinking any alcoholic beverage at least 12 times during the past year. All the subjects underwent three BP measurements in a sitting position with a 30 s interval after 5 minutes of rest, with electronic BP monitors (Omron HEM-770A, OMRON Healthcare Inc., Dalian, China). The mean of the three BP measurements was calculated for analysis. Waist circumference (WC) was measured at the level of 1 cm above the umbilicus. Body weight and height were measured with each subject wearing light clothing and without shoes. The body mass index (BMI) was calculated as weight in kilograms divided by square of the height in meters.

### 2.3. Follow-Up and Outcome Assessment

All participants were first followed up in 2013. Each participant was interviewed face to face by trained staff with a standard questionnaire. The investigated content was the same as that of the baseline investigation, also including histories of diseases and medication during follow-up. If a specific disease mentioned in the questionnaire was reported during the follow-up period, hospital records and experienced physicians also needed to be provided. Death data were confirmed by obtaining death certificates from the local civil registry or the attended hospital. The method and equipment for three BP measurements were the same as those used in the baseline survey. Hypertension was defined as systolic BP ≥ 140 mmHg and/or diastolic BP ≥ 90 mmHg and/or current use of antihypertensive medications.

### 2.4. Measurements

In the baseline investigation, blood samples were obtained in the morning by venipuncture after a requested overnight fasting period (at least 8 hours) and sampled in EDTA tubes and immediately spun at 3000 rpm for 15 minutes. Plasma samples were frozen at −80°C until laboratory testing, and measurements were performed by laboratory technicians who were blinded to the characteristics of the study patients. Total cholesterol (TC), triglycerides (TG), high-density lipoprotein cholesterol (HDL-C), and fasting plasma glucose (FPG) were analyzed enzymatically on a Hitachi 7020 automatic biochemical analyzer using commercial reagents (Kangxiang Medical Appliance, Shanghai, P.R China).

Plasma UII and ET-1 measurements were performed with each blood sample at baseline with enzyme-linked immunosorbent assay (ELISA) using standard kits (UII (human)-EIA kit, Phoenix Biotech, USA; ET-1 kit, R&D Systems China Co, Ltd) according to the manufacturer's instructions. Intra- and interassay coefficients of variation were both less than 4% and 8%.

### 2.5. Statistical Analysis

Due to skewed distribution, the concentration of ET-1 and other quantitative characteristics at baseline were described with the median and interquartile range (P_25_–P_75_). Independent Kruskal–Wallis rank tests were used to compare differences in these variables among four quartile groups according to the baseline UII level. Qualitative characteristics were presented as percentages and compared with the *χ*2 test. The characteristics were also presented with median (P_25_–P_75_) or absolute number (percentages) by the status of hypertension at the follow-up endpoint and were compared with *χ*2 test or Wilcoxon rank test. Four multiple logistic regression models were performed to analyze the association between baseline UII and the risk of hypertension with different combinations of confounder variables adjusted. The confounder variables including age, sex, systolic blood pressure, drinking, smoking, family history of hypertension, BMI, FPG, TC, and ET-1 were put into the four models from less to more. Participants were divided into four quartile groups according to the baseline UII levels and also were divided into two groups by the upper quartile of the baseline UII levels. The odds ratio (OR) and 95% confidence interval (CI) were calculated for the risk of hypertension associated with the baseline UII levels. A two-tailed *P* value less than 0.05 was considered statistically significant. All statistical analyses were conducted using SAS software (version 9.4, SAS Institute Inc., Cary, NC, USA).

## 3. Results

### 3.1. General Characteristics of Participants at Baseline

In the current population, there were 732 males and 1087 females. The average age at baseline was 51.6. There was no difference in levels of most characteristics except ET-1 at baseline among the quartile groups according to UII levels. The general characteristics of the participants at baseline are presented in Table 1 in detail.

### 3.2. General Characteristics at Baseline by Hypertension Status at the Follow-Up Point

Among the 1,819 subjects with normal blood pressure at baseline, 723 new cases of hypertension were found at the first follow-up point. Levels of ET-1 at baseline were significantly different between the new cases of hypertension and the normotensive participants, while levels of UII were not. The other general characteristics at baseline were compared between the new cases of hypertension and the normotensive participants (see Table 2).

### 3.3. Association between UII Level at Baseline and Risk of Hypertension

The participants were divided into four groups according to the quartiles of baseline UII levels to examine the prediction of UII at baseline on incident hypertension. ORs for risk of hypertension comparing the second quartile, the third quartile, and the highest quartile with the lowest quartile of baseline UII were not statistically significant in four models in which different combinations of confounding variables were adjusted. The confounding variables included age, sex, systolic blood pressure, drinking, smoking, family history of hypertension, BMI, FPG, TC, and ET-1. The detailed results are shown in Table 3.

We also pooled the lower three quartile groups as a reference and calculated ORs for risk of hypertension of the highest quartile of baseline UII. However, the ORs were not significant in the four logistic models. The results are listed in Table 4.

### 3.4. Analysis of the Association between Risk of Hypertension and UII Level at Baseline by Strata of ET-1 Levels

Furthermore, the subjects were divided into two groups by the 75th percentile (P_75_ = 1.56 pg/ml) of ET-1 levels. Then, we analyzed the association between the risk of hypertension and baseline UII in the subgroups. With the lower levels (<P_75_) of UII as a reference, the ORs and 95% CI of the higher levels of UII (≥P_75_) for hypertension were calculated in both the subgroups, and none of the ORs were significant with different combinations of confounder variables adjusted in the four logistic models. The detailed results are listed in Table 5.

## 4. Discussion

To our knowledge, this is the first prospective study to explore the association between plasma UII and the risk of hypertension. Our results showed that plasma UII was not associated with the risk of hypertension. Moreover, we did not find the effect of ET-1 on the association between plasma UII and the risk of hypertension. These indicated that plasma UII might not play a role in the development of hypertension. Our current study has a certain significance to demonstrate the association between plasma UII and the risk of hypertension with a large-scale prospective study. Some published studies showed that hypertension was associated with UII and indicated that it had an important significance in the treatment or prevention of hypertension. However, most of these studies were case-control studies, and the association was not verified in our prospective study. So further research is still needed to verify the association.

UII is a vasoconstrictor in rodent and primate animals. The vasoconstrictor activity depends on the race of animals, the position of vessels, and the status of vascular endothelium. The vasocontractile effect of UII acts by binding to the UII receptor (UT receptor). Ames et al. [14] found that UII existed widely in cardiovascular tissues and cloned the UT receptor, GPR14. UT receptor mRNA is widely expressed in human cardiovascular tissues, including cardiac myocytes, vascular smooth muscle cells, and endothelial cells. However, in rats, UT receptor mRNA was also expressed in motor neurons of the spinal cord, smooth muscle cells of the bladder, and cardiomyocytes [15]. The distribution patterns of the UII and UT receptor mRNA in man are not similar [16]. Maguire et al. [17] found that UII was approximately 50 times more potent than ET-1 in human arteries, while there was less than a ten-fold increase in potency of UII compared to ET-1 in human veins. MacLean et al. [18] found that UII was about four-fold more potent vasoconstrictor than ET-1 in rat main pulmonary arteries. And the response was increased by endothelial factors, vascular tone, and the onset of pulmonary hypertension, while inhibited by nitric oxide synthase. This evidence uncovers contractile responses to UII in human pulmonary arteries. Bottrill et al. [19] declared that UII was a potent endothelium-dependent vasodilator. However, most of these results were from animal studies or vitro studies and few population-based studies could verify these results.

Some clinical studies demonstrated that increased plasma UII levels were associated with the severity of carotid atherosclerosis and the severity of coronary artery lesions, such as in patients with essential hypertension [20] or patients with coronary heart disease [12]. Studies on the association between UII and hypertension were conducted mostly with the case-control study design. Some showed that levels of UII in plasma were higher in hypertensive than that in the control group [5, 6, 21–23]. Although these results indicated that UII might be a risk factor of hypertension, the relationship is not very certain for uncertain temporal relationships. In addition to this, several case-control studies showed that there was no significant difference in levels of plasma UII between normotensive and hypertensive patients [7, 8]. Even lower levels of plasma UII were found in hypertensive than in normotensive [24]. Zhou and Tian [25] compared levels of plasma UII among four groups with different levels of blood pressure, normal blood pressure, hypertension stage I, hypertension stage II, and hypertension stage III. They found no significant difference between the two groups of normal blood pressure and hypertension stage I. While higher levels in the group of hypertension stage II and hypertension stage III than in the normal group. The conclusion can be given that UII may drop out of the occurrence and development of hypertension.

As far as we know, there has been no other prospective cohort study on the association between UII and hypertension. We conducted the population-based study with about 2000 normotensive subjects and 5-year follow-up and found that levels of plasma UII at baseline were not associated with the risk of hypertension. Our result firstly exposed the association of UII and hypertension under the condition of clear temporal relationship.

Affolter et al. [26] conducted an intervention study with intravenous UII and saline placebo on ten healthy male volunteers and found no effect of intravenous UII infusion on systemic hemodynamics or arterial stiffness. They concluded that UII was unlikely to have a physiological role in the short-term regulation of vascular tone or blood pressure in man. Debiec and colleagues [27] studied the UII system in genetic control of blood pressure and renal function. No difference in renal expression of the UII system between normotensive and hypertensive subjects was found. This result suggested that UII system genes were unlikely to play a major role in the genetic control of human blood pressure or renal function. To some extent, the above two studies supported the results of our current study.

The association might be due to the condition of the endothelium and the modulation of the action of UII by endothelium-derived vasorelaxant factors such as nitric oxide [28]. Therefore, it is conceivable that the vasoconstricting effect of UII is brought to play in endothelial dysfunction. The results from subgroup analysis also indicated that there was no association between baseline UII level and risk of hypertension in different subgroups with different ET-1 levels. The subjects of our study were all with normal blood pressure at baseline, and endothelial dysfunction was unlikely to happen in these normotensive individuals. So our results indicated that UII may not participate in the development of hypertension.

The potential limitations of our study should be considered. Firstly, our current study is an observational study, which cannot confirm the causal relationship very powerfully. Further studies should be conducted, such as Mendelian randomization studies. Secondly, the vasodilatory actions of UII depend on the condition of the endothelium. However, we only examined levels of ET-1 in plasma to evaluate endothelial function in our study. Thirdly, levels of UII were examined only once at baseline in our current study. In consideration of the dynamic development of endothelial dysfunction and hypertension, two or more tests on UII and ET-1 at different time points should be performed, which may be better to explore the association between dynamic changes of levels of UII and ET-1 with the development of hypertension.

In summary, our study is the first one to explore the relationship between UII and risk of hypertension with a prospective cohort study and a large sample size. Our results indicate that UII is unlikely to play a role in the development of hypertension. Further confirmatory studies with UII system genetic expression based on population are required.

## Figures and Tables

**Table 1:** General characteristics of the 1819 participants by quartiles of levels of urotensin II at baseline.

	Quartiles of plasma urotensin II				*P* value
	Quartile 1 (*n* = 453)	Quartile 2 (*n* = 456)	Quartile 3 (*n* = 455)	Quartile 4 (*n* = 455)	
Age, year	52 (48–55)	52 (47–55)	52 (48–55)	51 (17–55)	0.386
Drinking, *n* (%)	103 (22.7)	96 (21.1)	88 (19.3)	91 (20.0)	0.612
Smoking, *n* (%)	135 (29.8)	139 (30.5)	143 (31.4)	148 (32.5)	0.827
Family history of HTN, *n* (%)	187 (41.3)	223 (48.9)	201 (44.2)	208 (45.7)	0.135
Body mass index (kg/m^2^)	22.5 (20.4–25.0)	22.8 (20.2–25.1)	22.4 (20.6–24.1)	22.5 (20.5–24.6)	0.699
Waist circumference (cm)	80 (74–87)	80 (73–86)	80 (73–85)	79 (72–85)	0.100
Systolic blood pressure (mmHg)	122 (115–130)	121 (113–130)	120 (112–128)	121 (113–129)	0.268
Diastolic blood pressure (mmHg)	77 (72–82)	77 (71–82)	75 (69–81)	76 (71–82)	0.210
Fasting plasma glucose (mmol/L)	4.9 (4.6–5.2)	4.9 (4.5–5.2)	4.8 (4.4–5.2)	4.8 (4.4–5.2)	0.043
Total cholesterol (mmol/L)	4.5 (4.0–5.1)	4.5 (3.9–5.1)	4.5 (4.0–5.2)	4.5 (3.9–5.1)	0.830
Triglycerides (mmol/L)	1.2 (0.9–1.9)	1.3 (1.1–1.5)	1.3 (0.9–1.9)	1.3 (1.0–1.5)	0.225
HDL-cholesterol (mmol/L)	1.3 (1.1–1.5)	1.3 (1.1–1.5)	1.3 (1.1–1.5)	1.3 (1.1–1.5)	0.052
LDL-cholesterol (mmol/L)	2.5 (2.0–3.0)	2.4 (2.0–2.9)	2.5 (2.0–3.0)	2.5 (2.1–3.0)	0.389
Endothelin-1 (pg/ml)	1.21 (0.86–1.68)	1.09 (0.80–1.54)	1.04 (0.73–1.48)	1.17 (0.81–1.57)	<0.0001

Numerical variables were expressed as median (P_25_–P_75_); HTN: hypertension; HDL: high-density lipoprotein; LDL: low-density lipoprotein.

**Table 2:** Comparison of baseline characteristics between the new cases of hypertension and the normotensive participants.

	Normotensive (*n* = 1096)	Hypertensive (*n* = 723)	*P* value
Age, year	51 (47–54)	53 (49–56)	<0.0001
Drinking, *n* (%)	218 (19.9)	160 (22.1)	0.249
Smoking, *n* (%)	351 (32.0)	214 (29.6)	0.274
Family history of HTN, *n* (%)	465 (42.4)	354 (49.0)	0.006
Body mass index (kg/m^2^)	21.9 (20.0–24.1)	23.4 (21.1–25.7)	0.699
Waist circumference (cm)	76 (70–82)	80 (73–86)	0.100
Systolic blood pressure (mmHg)	116 (109–126)	128 (121–134)	<0.0001
Diastolic blood pressure (mmHg)	74 (68–79)	81 (76–85)	<0.0001
Fasting plasma glucose (mmol/L)	4.8 (4.4–5.1)	4.9 (4.6–5.3)	0.043
Total cholesterol (mmol/L)	4.4 (3.9–5.0)	4.6 (4.1–5.3)	0.830
Triglycerides (mmol/L)	1.2 (0.9–1.7)	1.4 (1.0–1.5)	0.225
HDL-cholesterol (mmol/L)	1.3 (1.1–1.5)	1.3 (1.1–1.5)	0.052
LDL-cholesterol (mmol/L)	2.4 (2.0–3.0)	2.5 (2.1–3.0)	0.389
Endothelin-1 (pg/ml)	1.12 (0.78–1.57)	1.15 (0.80–1.53)	<0.0001
Urotensin II (pg/ml)	83.37 (65.91–109.45)	80.63 (64.11–105.62)	0.203

Numerical variables were expressed as median (P_25_–P_75_); HTN, hypertension; HDL, high-density lipoprotein; LDL, low-density lipoprotein.

**Table 3:** Odds ratios for risk of hypertension comparing the three higher quartiles with the lowest quartile of baseline urotensin II levels.

	Quartile of plasma urotensin II				*P* value for trend
	Quartile 1 (*n* = 453)	Quartile 2 (*n* = 456)	Quartile 3 (*n* = 455)	Quartile 4 (*n* = 455)	
Number of new cases of HTN (%)	188 (41.5)	190 (41.7)	173 (38.0)	172 (37.8)	
Model 1^a^	1.000	1.008 (0.772–1.317)	0.848 (0.647–1.110)	0.870 (0.665–1.140)	0.573
Model 2^b^	1.000	1.119 (0.829–1.511)	1.046 (0.774–1.414)	0.930 (0.688–1.256)	0.910
Model 3^c^	1.000	1.096 (0.811–1.481)	1.037 (0.766–1.403)	0.923 (0.683–1.248)	0.948
Model 4^d^	1.000	0.993 (0.753–1.310)	0.860 (0.650–1.140)	0.879 (0.664–1.163)	0.775

^a^Adjusted for age and sex; ^b^adjusted for age, sex, and systolic blood pressure at baseline; ^c^adjusted for age, sex, systolic blood pressure, drinking, smoking, and family history of hypertension; ^d^adjusted for age, sex, systolic blood pressure, drinking, smoking, family history of hypertension, body mass index, fasting plasma glucose, total cholesterol, and endothelin-1; HTN, hypertension.

**Table 4:** Odds ratios of the risk of hypertension associated with baseline urotensin II levels in two categories.

	Odds ratio (95% confidence interval)	
	Quartiles 1–3 (*n* = 1364)	Quartile 4 (*n* = 455)
Number of new cases of HTN (%)	551 (40.4)	172 (37.8)
Model 1^a^	1.000	0.917 (0.747–1.094)
Model 2^b^	1.000	0.883 (0.689–1.131)
Model 3^c^	1.000	0.885 (0.690–1.135)
Model 4^d^	1.000	0.888 (0.689–1.144)

^a^Adjusted for age and sex; ^b^adjusted for age, sex, and systolic blood pressure at baseline; ^c^adjusted for age, sex, systolic blood pressure, drinking, smoking, and family history of hypertension; ^d^adjusted for age, sex, systolic blood pressure, drinking, smoking, family history of hypertension, body mass index, fasting plasma glucose, total cholesterol, and endothelin-1; HTN, hypertension.

**Table 5:** Odds ratios of risk of hypertension associated with baseline urotensin II levels by strata of endothelin-1 levels.

	Endothelin-1 <1.56 pg/ml (*n* = 1357)		Endothelin-1 ≥1.56 pg/ml (*n* = 452)	
	Urotensin II < P_75_	Urotensin II ≥ P_75_	Urotensin II < P_75_	Urotensin II ≥ P_75_
Model 1^a^	1.000	0.914 (0.707–1.180)	1.000	0.928 (0.595–1.449)
Model 2^b^	1.000	0.847 (0.635–1.129)	1.000	1.012 (0.609–1.684)
Model 3^c^	1.000	0.846 (0.634–1.129)	1.000	1.027 (0.615–1.712)
Model 4^d^	1.000	0.866 (0.647–1.159)	1.000	0.987 (0.587–1.660)

^a^Adjusted for age and sex; ^b^adjusted for age, sex, and systolic blood pressure at baseline; ^c^adjusted for age, sex, systolic blood pressure, drinking, smoking, and family history of hypertension; ^d^adjusted for age, sex, systolic blood pressure, drinking, smoking, family history of hypertension, body mass index, fasting plasma glucose, and total cholesterol.

## Data Availability

The data used to support the findings of this study were supplied by Mingzhi Zhang under license and so cannot be made freely available. Requests for access to these data should be made to Mingzhi Zhang, zhangmingzhi@suda.edu.cn.
